# Pooling individual participant data from randomized controlled trials: Exploring potential loss of information

**DOI:** 10.1371/journal.pone.0232970

**Published:** 2020-05-12

**Authors:** Lennard L. van Wanrooij, Marieke P. Hoevenaar-Blom, Nicola Coley, Tiia Ngandu, Yannick Meiller, Juliette Guillemont, Anna Rosenberg, Cathrien R. L. Beishuizen, Eric P. Moll van Charante, Hilkka Soininen, Carol Brayne, Sandrine Andrieu, Miia Kivipelto, Edo Richard

**Affiliations:** 1 Department of Neurology, Amsterdam UMC, University of Amsterdam, Amsterdam, The Netherlands; 2 Department of Neurology, Donders Institute for Brain, Cognition and Behaviour, Radboud University Medical Center, Nijmegen, The Netherlands; 3 Department of Epidemiology and Public Health, Toulouse University Hospital, Toulouse, France; 4 INSERM, University of Toulouse UMR1027, Toulouse, France; 5 Chronic Disease Prevention Unit, National Institute for Health and Welfare, Helsinki, Finland; 6 Department of Information and Operations Management, ESCP Europe, Paris, France; 7 INSERM, University of Toulouse, Toulouse, France; 8 Department of Neurology, Institute of Clinical Medicine, University of Eastern Finland, Kuopio, Finland; 9 Department of General Practice, Amsterdam UMC, University of Amsterdam, Amsterdam, The Netherlands; 10 Neurocenter, Neurology, Kuopio University Hospital, Kuopio, Finland; 11 Department of Public Health and Primary Care, Cambridge Institute of Public Health, University of Cambridge, Cambridge, United Kingdom; 12 Aging Research Center, Karolinska Institutet, Stockholm University, Stockholm, Sweden; 13 Karolinska Institutet Center for Alzheimer Research, Stockholm, Sweden; Universitat Bern, SWITZERLAND

## Abstract

**Background:**

Pooling individual participant data to enable pooled analyses is often complicated by diversity in variables across available datasets. Therefore, recoding original variables is often necessary to build a pooled dataset. We aimed to quantify how much information is lost in this process and to what extent this jeopardizes validity of analyses results.

**Methods:**

Data were derived from a platform that was developed to pool data from three randomized controlled trials on the effect of treatment of cardiovascular risk factors on cognitive decline or dementia. We quantified loss of information using the R-squared of linear regression models with pooled variables as a function of their original variable(s). In case the R-squared was below 0.8, we additionally explored the potential impact of loss of information for future analyses. We did this second step by comparing whether the Beta coefficient of the predictor differed more than 10% when adding original or recoded variables as a confounder in a linear regression model. In a simulation we randomly sampled numbers, recoded those < = 1000 to 0 and those >1000 to 1 and varied the range of the continuous variable, the ratio of recoded zeroes to recoded ones, or both, and again extracted the R-squared from linear models to quantify information loss.

**Results:**

The R-squared was below 0.8 for 8 out of 91 recoded variables. In 4 cases this had a substantial impact on the regression models, particularly when a continuous variable was recoded into a discrete variable. Our simulation showed that the least information is lost when the ratio of recoded zeroes to ones is 1:1.

**Conclusions:**

Large, pooled datasets provide great opportunities, justifying the efforts for data harmonization. Still, caution is warranted when using recoded variables which variance is explained limitedly by their original variables as this may jeopardize the validity of study results.

## Introduction

The sample size in individual cohort studies and randomized controlled trials (RCTs) is often too small to answer specific (secondary) research questions. Pooling individual participant data (IPD) from multiple studies increases the sample size and statistical power to perform subgroup analyses and enable assessment of consistency of findings across different studies. To enable IPD meta-analyses it is necessary to harmonize data from different studies, which is often complicated by diversity across the available datasets.

Differences in data collection include, but are not limited to, data in multiple file formats and languages, the use of different instruments or scales to measure the same domains and use of different units of measurement. When syntaxes to recode data are developed ad hoc, without detailed information on the data collection procedure, misinterpretation may lead to a decrease of validity of the data [1].

For this reason much attention is given to state-of-the-art data harmonization techniques, as evidenced by the development of software such as DataSHAPER and ViPAR [2, 3]. These initiatives facilitate data pooling from different studies while overcoming differences in the collection of the data. Regardless of whether data harmonization is performed using specific software or by hand, little is known on the extent of information that is lost during the data harmonization process.

The aim of this study was to quantify the consequences of recoding variables for data pooling with regards to loss of information and the resulting loss of validity of study results. We therefore explored how much information was lost after recoding of variables, expressed as the proportion of the variance in the pooled variables that is explained by the data in the original datasets. We hypothesized most variance was lost when variables were recoded from continuous to discrete, which is in line with previous studies. We used data that were pooled from three clinical trials as well as simulated data to study our hypothesis. When a substantial loss of information occurred for a non-simulated variable, we additionally explored the potential impact on the validity of analyses outcomes. In a simulation study, we explored the influence of the range of continuous variables and ratio of dichotomous variables on information loss. Finally, we share all recoding schemes that we used for pooling variables relevant for research on cardiovascular risk factors and cognitive decline.

## Methods

### Data collection

Individual participant data from three recently completed RCTs on multi-domain interventions to prevent cognitive decline or dementia with a total of 6435 participants, were pooled. These were the Prevention of Dementia by Intensive Vascular Care trial (preDIVA, ISRCTN 29711771 [4]), the Finnish Geriatric Intervention Study to Prevent Cognitive Impairment and Disability trial (FINGER, NCT 01041989 [5]) and the Multidomain Alzheimer Preventive Trial (MAPT, NCT 00672685 [6]). The study teams of these three clinical trials collaborate in the HATICE consortium (www.hatice.eu), and are dedicated to share data amongst each other. All data had been pseudonymized prior to access for analysis. In Table 1 the study characteristics are shown.

**Table 1:** Main study characteristics of pooled studies.

	preDIVA	FINGER	MAPT
**Year started**	2006	2009	2008
**Year completed**	2015	2014	2014
**Sample size**	3526	1260	1679
**Age (years)**	70–78	60–75	>70
**Intervention (years)**	6–8	2	3
**Primary outcome**	dementia incidence, disability level	change in cognitive function	change in cognitive function
**Main secondary outcomes**	cardiovascular events, change in cognitive function, depression	cardiovascular events, dementia incidence, depression, disability level, quality of life, health resources utilization	cardiovascular events functional assessment, depression, dementia incidence, health resources utilization

### Recoding schemes

The pooled dataset consisted of 170 variables, of which 101 were available for the original FINGER subset of the database, 137 for the MAPT subset and 91 for the preDIVA subset. This sums to a total 329 recoding schemes that were necessary to create the pooled variables. For 238 recoding schemes an algorithmic transformation of the original variable was not necessary to create the pooled variable. We did not perform analyses for these recoding schemes, since these would be redundant by definition. This means we conducted analyses only for those 91 recoding schemes in which an algorithmic transformation of the original variable(s) was required to create the pooled variable. Three different algorithmic transformations were used: (1) Continuous variables that were recoded into discrete variables (N_original_ = 8); (2) Discrete variables that were recoded into discrete variables with a different number or order of categories (N_original_ = 44); and (3) Recoded variables that were based on multiple original variables (N_original_ = 39).

### Statistical analyses

**Fig 1:**
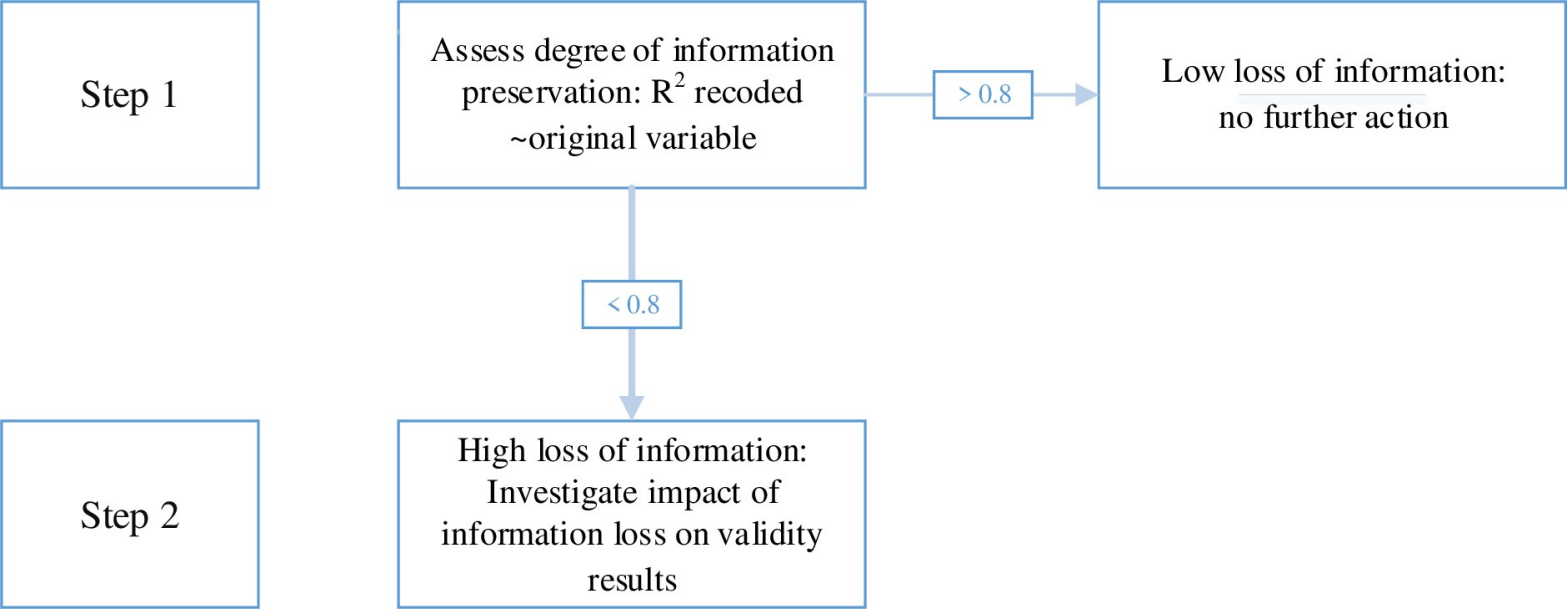
Flowchart of step 1 and step 2 analyses.

For the current analyses we focus on available baseline data. Handling missing data with context-free data encoding has been described previously [7, 8]. We used a stepwise procedure to first quantify the information loss of pooled variables, and subsequently explored the potential impact on future analyses by using variables that had lost most information (Fig 1).

Step 1: We used linear regression models with the recoded variables as dependent variables and the original variables as independent variables and extracted the R-squared as measures for explained variance. In case a recoded variable was based on multiple original variables, we used these as predictors in a single linear regression model. When the R-squared was at least 0.8, an arbitrarily set threshold, we considered the amount of information that was lost acceptable, else we continued with step 2.

Step 2: To explore the potential impact of the information loss, in a linear regression model we assessed whether the Beta coefficient of an independent variable changed by more than 10% when using the pooled variable instead of the original variable as a confounder. A Beta coefficient is the degree of change in the dependent variable for each one unit increase of the independent variable [9]. This is analogous to commonly applied criteria for confounders [10]. For these regression models we chose an independent and dependent variable which we, based on literature, expected to be associated to each other as well as to the confounder. Therefore, we used various independent and dependent variables for these step 2 analyses.

In our simulation study we randomly generated continuous variables and recoded these to dichotomous variables. For these simulated variables we explored how much information was lost with varying range of the continuous variable (type 1a), varying ratio of recoded ones to recoded zeroes (type 1b) and a combination of both (type 2). For all simulations we sampled two sets of numbers. The sampling method for the first set was the same across iterations, while the second set differed in range of continuous numbers, ratio of recoded ones to recoded zeroes (by differing sample sizes recoded to 1 while the amount recoded to 0 remained the same), or both. For the first set 1000 numbers between 0 and 1000 were sampled. For the second set we sampled K numbers between 1001 and N. For type 1a K was kept at a constant of 1000, while N was a factor of between 1 and 34 as high or low compared to 1000. Therefore, the lowest N was 1030 (1000/34+1001) and the highest N was 35001 (1000*34+1001). For type 1b N was kept at a constant of 1000, while K was a factor of between 1 and 34 as high or low compared to 1000. The lowest K was therefore 29 (1000/34) and the highest K 34000 (1000*34). For type 1a and 1b each of the 67 simulations (a factor of 1 as high or low is the same) was replicated 100 times. For our type 2 simulations, all combinations of K and N from type 1a and 1b were used, therefore yielding 4489 (67 * 67) combinations. Each of these combinations was replicated 10 times. With this latter simulation type we tested to what extent information loss caused by a difference in range for set 1 and set 2 numbers can be compensated for or exaggerated by varying the ratio of ones to zeroes. For all three types of simulations numbers between 0 and 1000 were recoded to 0 and those above 1000 to 1. We then could use the same linear models as in the Step 1 analyses, in which the recoded zeroes and ones were the dependent variables and the sampled numbers the independent variables. From these models we again extracted the R-squared to explore to what extent the R-squared changed depending on the difference in range of numbers, the ratio of ones to zeroes or a combination of both. The full syntax of this simulation is available in the supplement.

Normality and homoscedasticity of residuals have been checked for the linear regression models. The impact of violations of assumptions are discussed at the end of the results section. All analyses have been conducted using R Studio [11], specifically the built-in package ‘stats’ [12] for the linear models and the additionally loaded packages ‘ggplot2’ [13] and ‘gridExtra’ [14] for the visualizations.

Participants gave written informed consent prior to their baseline visit. The preDIVA study was approved by the Medical Ethics Committee of the Academic Medical Center, Amsterdam. The MAPT trial protocol was approved by the French Ethical Committee located in Toulouse (CPP SOOM II) and was authorized by the French Health Authority. FINGER was approved by the coordinating ethics committee of the Hospital District of Helsinki and Uusimaa.

## Results

**Fig 2:**
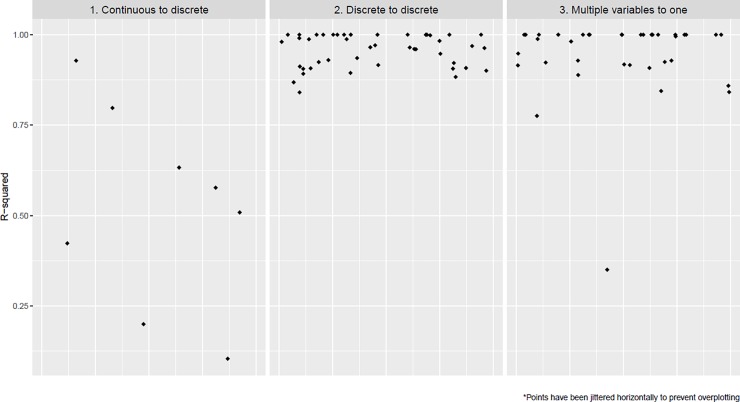
Pooling accuracy for three data recoding categories.

For the eight continuous-to-discrete recoded variables, the median R-squared of the regression models, as described as the first step of our analyses, was 0.54 (IQR: 0.37–0.67). For the 44 discrete-to-discrete recoded variables it was 0.97 (IQR: 0.92–1.00) and for the 39 multiple-to-single recoded variables 0.98 (IQR: 0.92–1.00) (Fig 2). All individual R-squareds and the recoding schemes, listed by content category, are provided in the S1 File.

Eight regression models yielded an R-squared of below 0.8. For these we performed exploratory analyses as described for our step 2 method (Table 2). The Beta coefficient of the independent variable changed by more than 10% in four out of eight models depending on whether recoded or original confounders were used. In other words, in half of the models the Beta coefficient changed considerably when using the recoded instead of original variables as confounders, which may lead to a different interpretation of the results.

**Table 2:** Change of the beta coefficient of an association when using the recoded variable as a confounder compared to the original variable for the variables with less than 80% explained variance after recoding to assess the impact of information loss on the validity of associations.

Original variable	Recoded variable	Study	Proportion of variance explained	Dependent variable	Independent variable	Βeta of independent variable when using original confounder	Βeta of independent variable when using recoded confounder	Change in Beta >10%
Number of years stopped smoking	Stopped smoking more than 3 years (yes/no)	preDIVA	0.10	BMI^a^	Age	-0.027 (-0.098 to 0.043, p = 0.448)	-0.042 (-0.112 to 0.028, p = 0.237)	yes
Glucose level (mmol/L)	Glucose normal (yes/no)	preDIVA	0.20	Diagnosis of diabetes	LDL^b^	-0.076 (-0.086 to -0.065, p < .001)	-0.125 (-0.138 to -0.112, p < .001)	yes
Glucose level (g/L)	Glucose normal (yes/no)	MAPT	0.35	Diagnosis of diabetes	LDL	-0.038 (-0.085 to 0.009, p = 0.111)	-0.045 (-0.090 to 0.000, p < .001)	yes
Zung^c^ sumscore	Depression (yes/no)	FINGER	0.42	MMSE^d^ sum score	Age	-0.043 (-0.068 to -0.017, p = 0.001)	-0.045 (-0.070 to -0.019, p = 0.001)	no
Glucose level (mmol/L)	Glucose normal (yes/no)	FINGER	0.51	Diagnosis of diabetes	LDL	-0.083 (-0.102 to -0.064, p < .001)	-0.084 (-0.102 to -0.065, p < .001)	no
GDS-15^e^ sum score	Depression	preDIVA	0.58	MMSE sum score	Age	-0.016 (-0.040 to 0.007, p = 0.179)	-0.020 (-0.045 to 0.004, p = 0.107)	yes
GDS-15 sum score	Depression	MAPT	0.63	MMSE sum score	Age	-0.047 (-0.064 to -0.030, p < .001)	-0.048 (-0.066 to -0.031, p < .001)	no
Heart disease: Father (yes/no) Mother (yes/no) Sibling (yes/no) Child (yes/no)	Family history of heart disease (yes/no)	preDIVA	0.78	History of heart disease (yes/no)	LDL	-0.130 (-0.145 to -0.114, p < .001)	-0.130 (-0.145 to -0.114, p < .001)	no

^a^ Body mass index

^b^ low-density lipoprotein

^c^ Zung Self-Rating Depression Scale

^d^ Mini-Mental State Examination

^e^ Geriatric Depression Scale

**Fig 3:**
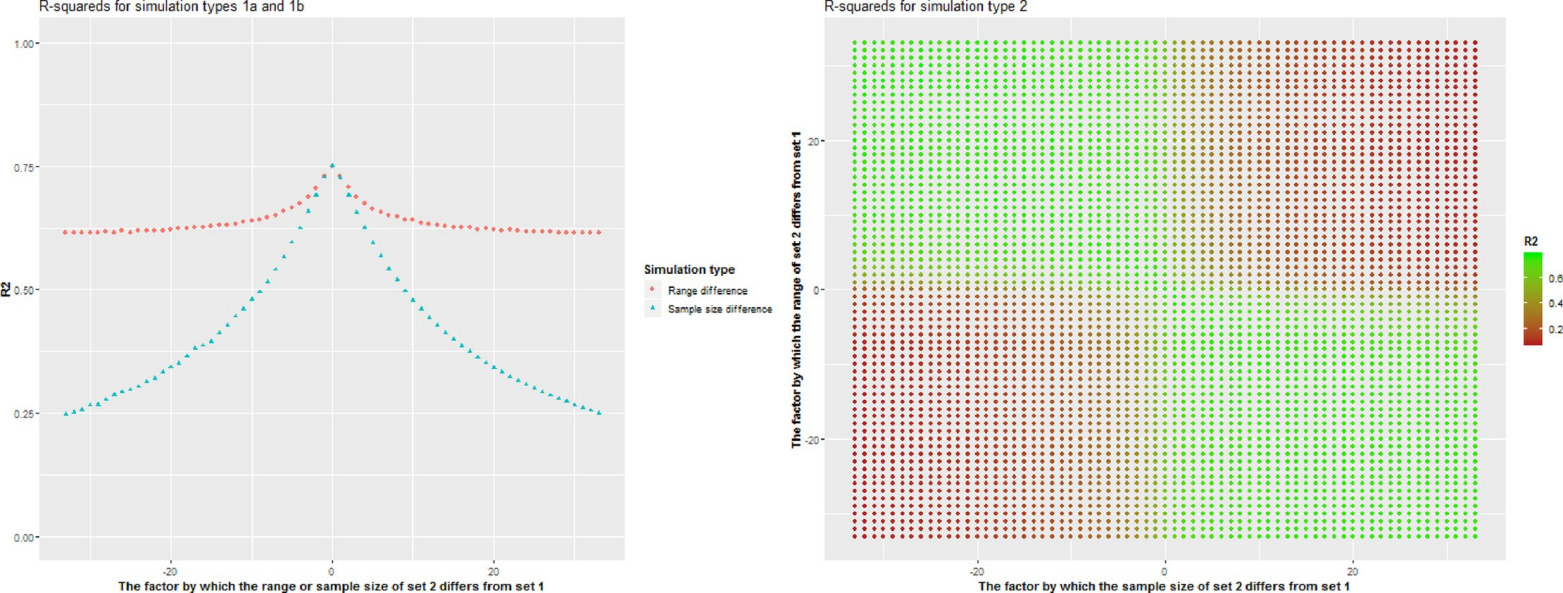
R2s for simulations in which numbers between 0 and 1000 are recoded to 0 and those above 1000 to 1. For all iterations, 1000 numbers between 0 and 1000 were sampled and recoded to 0. Numbers that were recoded to 1 originated from sampling numbers between 1001 and N with sample size K. Left: K was a constant of 1000, N was between 1 and 34 times as high or low as 1000 (simulation type 1a, red circles); N was a constant of 2001, K was between 1 and 34 times as high or low as 1000 (simulation type 1b, blue triangles). Right: N was between 1 and 34 times as high or low as 1000 and K was between 1 and 34 times as high or low as 1000 (simulation type 2).

The type 1a simulation study, in which the range of numbers recoded to 1 differed from the range of numbers recoded to 0, yielded a median R-squared of 0.63 (IQR: 0.62–0.64). The median R-squared of the type 1b simulation, in which the ratio of ones to zeroes varied, was 0.38 (IQR: 0.30–0.51). This means the R-squared is more negatively impacted by differences in amount of ones compared to zeroes than by difference in ranges between numbers that were either recoded to 0 or 1. When combined, in type 2 of our simulation study, the median R-squared was 0.54 (IQR: 0.16–0.74). When the range of the set 2 numbers increased, the R-squared increased when the ratio of ones to zeroes decreased, while the R-squared decreased even more when the ratio of ones to zeroes also increased (Fig 3).

For most linear regression models the assumptions of normality and homoscedasticity of residuals were met. We exploratively assessed to what extent the R-squared changed when log-transformed dependent and independent variables were used instead of untransformed variables. We did this for all 91 linear regression models in which pooled variables were modelled that had not been pooled directly. We observed a median change of 0.03 (IQR: 0.00–0.06) and a maximum change of 0.27 in the R-squared when this procedure was used. This suggests using log-transformed variables for this purpose generally does not considerably alter the R-squared.

## Discussion

In pooling data from three RCTs, recoding of 91 variables resulted in a loss of explained variance of more than 20% in 8 variables. Most substantial loss was observed for variables that were recoded from continuous to discrete. Exploratory analysis suggested that the impact of recoded variables with substantial loss of explained variance on multivariate analyses might not be trivial.

To our knowledge, this study is the first to explore the degree of information in original data that is lost when harmonizing data, including analyses to assess potential consequences for the validity of findings using recoded variables from a pooled dataset. Although most of the recoded variables within the harmonized database appear to be valid and reliable, those variables that lost a substantial part of the explained variance following their recoding should perhaps be left out of future analyses or be handled with caution. These include important areas of study that are measured as continuous such as cognitive and depressive symptomatology.

Using data dictionaries such as CDISC when designing studies is recommended. Within a specific research field, a certain level of harmonization of assessment instruments would also reduce the need for recoding. However, even if agreement will be attained, recoding variables is sometimes inevitable, for example when pooling data from trials from countries with different standard measures. Specific attention should be paid to assessing of the potential altered findings when analyzing data with variables that were recoded from a continuous to a discrete scale. We encourage other researchers conducting pooled analyses to include a quantification of the information that was lost due to the recoding process. Also, we recommend that when data has been recoded for a pooled analysis, the analysis should be repeated in the original dataset with both the original and the recoded variable, and results should be reported (at least in a supplement), to illustrate the impact of recoding. Using the R-squared of linear regression models is appropriate as a crude summary to quantify information loss of pooled variables. When the dependent variable is nominal, a linear regression may not be the appropriate analysis. For consistency and enhancement of comparability of the R-squareds as a crude summary of information loss, we decided to use linear regression models for all types of recoded variables. More generally, this method may be less valid in case of violations of assumptions for linear models. However, exploratory analysis showed that using log-transformed variables instead of untransformed variables has only limited impact on the R-squared (median change in R-squared after transformation of all 91 non-directly pooled models: 0.03 (IQR: 0.00–0.06)). Adding the R-squared of key variables in pooled analyses in summary results allows readers to assess the full impact of harmonization of data on research findings.

The impact of data harmonization is most substantial in case a continuous variable is recoded into a dichotomous variable, as hypothesized. This in line with findings of previous studies [15, 16] and followed both from our main analyses as well from our simulation study. Our different types of simulations showed that the R-squared is more influenced by differences in ratio of ones to zeroes than by differences in ranges between numbers that have been recoded to 0 or 1. Also, it followed that increasing differences in ranges between two sets, can be compensated for by decreasing the size difference between the two groups of numbers that have been recoded to either 0 or 1, and vice versa. We do not recommend excluding variables that have been recoded from continuous to dichotomous from pooled analyses for the purpose of increasing overall explained variance of pooled variables.

To conclude, large, pooled datasets provide important opportunities, justifying the efforts for data harmonization. However, caution is warranted when using recoded variables whose variance is poorly related to that of the original variables as this may jeopardize the validity of study results.

## Supporting information

S1 File Recoding schemes and R-squareds for all pooled variables. (DOCX)
