# Supplementary material for: Pooling individual participant data from randomized controlled trials: Exploring potential loss of information
Source: PLoS One. 2020 May 12;15(5):e0232970. doi: 10.1371/journal.pone.0232970 (PMC7217432; doi:10.1371/journal.pone.0232970)
Supplement: S1 File — (DOCX) [file pone.0232970.s001.docx]

**Electronic supplementary material to “Pooling individual participant data from randomized controlled trials: Exploring potential loss of information.”**Recoding schemes and R-squareds for all pooled variables

This supplement contains 7 tables with recoding schemes. In the far left column the pooled variables are shown, followed by the recoding schemes we applied on the original variables of the MAPT, FINGER and preDIVA datasets and the R-squareds that resulted from the linear regression models (with the pooled variables as dependent variables and original variables as independent variables). Each table reports on one category of variables: 1) Biology and anthropometry; 2) Functional assessment; 3) Identification variables; 4) Cardiovascular lifestyle; 5) Medical history; 6) Neuropsychological assessment; 7) Sociodemographic characteristics.

In addition, in Table 8 missing percentages per variable and study are shown and in the 9^th^ section we show the R syntax we used for our simulation study.

**Abbreviations:**

- **DP:** Directly pooled
- **DtD:** Discrete to discrete with a different number or order of categories
- **CtD:** Continuous to discrete
- **MtS:** Multiple variables into a single variable
- **NA:** Variable not available in this original study
- **R2:** R-squared
- **orig.:** original
- **MAPT:** Multidomain Preventive Alzheimer Trial
- **FINGER:** Finnish Geriatric Intervention Study to Prevent Cognitive Impairment and Disability trial
- **preDIVA:** Prevention of Dementia by Intensive Vascular Care trial

**1. Biology and anthropometry**

| **Pooled variable** | **MAPT orig. vars.** | **MAPT R2’s** | **FINGER orig. vars** | **FINGER R2’s** | **preDIVA orig. vars** | **preDIVA R2’s** |
| --- | --- | --- | --- | --- | --- | --- |
| Weight in kilograms | DP | 1 | DP | 1 | DP | 1 |
| Height in centimeters | DP | 1 | DP | 1 | DP | 1 |
| Body Mass Index (BMI) | DP | 1 | DP | 1 | DP | 1 |
| Waist | DP | 1 | DP | 1 | DP | 1 |
| Systolic blood pressure | DP | 1 | DP | 1 | DP | 1 |
| Diastolic blood pressure | DP | 1 | DP | 1 | DP | 1 |
| Pulse | DP | 1 | DP | 1 | DP | 1 |
| Cholesterol | DP | 1 | DP | 1 | DP | 1 |
| High-density lipoprotein (HDL) | DP | 1 | DP | 1 | DP | 1 |
| Low-density lipoprotein (LDL) | DP | 1 | DP | 1 | DP | 1 |
| Triglycerides | DP | 1 | DP | 1 | DP | 1 |
| Non-fasting glucose level | If fasting = ‘no’:   - If glucose level is measured on g/L scale, glucose level multiplied by 5.5; - If glucose level is measured on mmHg/L scale, no multiplication needed   If fasting = ‘yes’: missing | 1 | NA | - | DP | 1 |
| Fasting glucose level | If fasting = yes’:   - If glucose level is measured on g/L scale, glucose level multiplied by 5.5; - If glucose level is measured on mmHg/L scale, no multiplication needed   If fasting = ‘no’: missing | 1 | DP | 1 | NA | - |
| Glucose level normal: | MtS | 0.35 | CtD | 0.51 | CtD | 0.20 |
| - *No* | - *Non-fasting glucose ≥ 11.1 or fasting glucose ≥7* |  | - *Fasting glucose ≥7* |  | - *Non-fasting glucose ≥ 11* |  |
| - *Yes* | - *Non-fasting glucose < 11.1 or fasting glucose <7* |  | *Fasting glucose <7* |  | - *Non-fasting glucose < 11* |  |
| Creatinine | DP | 1 | DP | 1 | DP | 1 |
| CRP | NA | - | DP | 1 | DP | 1 |
| ApoA1 | NA | - | DP | 1 | DP | 1 |
| ApoB | NA | - | DP | 1 | DP | 1 |

**2. Functional assessment**

| **Pooled variable** | **MAPT orig. vars.** | **MAPT R2’s** | **FINGER orig. vars** | **FINGER R2’s** | **preDIVA orig. vars** | **preDIVA R2’s** |
| --- | --- | --- | --- | --- | --- | --- |
| Home support: | MtS | 0.92 | DtD | 0.96 | NA | - |
| - *0. Never* | - *Home support = ‘no’* |  | - *Not at all* |  |  |  |
| - *1. Once a week or less* | - *Home support = ‘yes’ and total hours < 1.5* |  | - *Occasionally to once a week* |  |  |  |
| - *2. 2-4 times a week* | - *Home support = ‘yes’ and 1.5 > total hours < 4.5* |  | - *2-4 times a week* |  |  |  |
| - *3. 5 times a week or more* | - *Home support = ‘yes’ and total hours ≥ 1.5* |  | - *More than once a day or daily or almost daily* |  |  |  |

For all other variables related to functional assessment for MAPT we used items from the Activities of Daily Living (ADL) and Alzheimer’s Disease Cooperative Study Activities of Daily Living Prevention Instrument (ADCS-ADL-PI) questionnaires [1, 2]. For preDIVA the AMC Linear Disability Scale (ALDS) has been used [3]. The ADL questions were specific for each functional domain, while the ADCS-ADL had the following general form: *Please tick the one box that best describes how you have been doing each activity in the past 3 months (3. As well as usual, with no difficulty; 2. With a little difficulty; 1. With a lot of difficulty; 0. Did not do this activity.)* The ALDS had the general form: *Can you…? (0. Yes; 1. Yes with some effort; 2. No; 3. Unknown)* The questions used for FINGER were derived from basic ADL and instrumental ADL questionnaires, which had the following general form: *How do you manage following activities of daily living? (1. I can without difficulties; 2. Some difficulties; 3. Great difficulties; 4. Needs assistance; 5. Unable to manage) [1, 4, 5]*

The following general recoding procedure was used to obtain the pooled variables. The general question form of the pooled variables was: *Are you able to perform the following activities? (0. No; 1. With difficulties; 2. Yes)*

| **New pooled variable values** | **MAPT** | | **FINGER** | **preDIVA** |
| --- | --- | --- | --- | --- |
|  | ADL | ADCS-ADL-PI | ADL | ALDS |
| 0. No | 0. Lowest score | 0. You did not perform this activity | 5. Unable to manage  4. Needs assistance | 2. No |
| 1. With difficulties | 0.5. Intermediate score | 1. With great difficulty  2. With some difficulty | 3. Great difficulties  2. Some difficulties | 1. Yes with some effort |
| 2. Yes | 1. Higher score | 3. As well as usual, without difficulty | 1. I can without difficulties | 0. Yes |
| Missing |  |  |  | 3. Unknown |

Six variables were recoded twice, also to pooled variables with four possible values. For these the following recoding procedure was used for MAPT and FINGER.

| **New pooled variable values** | **MAPT** | **FINGER** |
| --- | --- | --- |
|  | ADCS-ADL | ADL |
| 0. No | 0. You did not perform this activity | 5. Unable to manage  4. Needs assistance |
| 1. With great difficulties | 1. With great difficulty | 3. Great difficulties |
| 2. With some difficulties | 2. With some difficulty | 2. Some difficulties |
| 3. Yes | 3. As well as usual, without difficulty | 1. I can without difficulties |

The “able to prepare food” and “able to do light housework” pooled variables were derived from preDIVA by combining two ALDS items. To obtain the 3-value variables the following procedure was used:

| ALDSx  ALDSy | 0. Yes | 1. Yes with some effort | 2. No | 3. Unknown |
| --- | --- | --- | --- | --- |
| 0. Yes | 2. Yes | 1.With difficulties | 1. With difficulties | Missing |
| 1. Yes with some effort | 1. With difficulties | 1.With difficulties | 1. With difficulties | Missing |
| 2. No | 1. With difficulties | 1.With difficulties | 0. No | Missing |
| 3. Unknown | Missing | Missing | Missing | Missing |

At last, to create the 4-value variables for “able to prepare food” and “able to do light housework” the following procedure was used:

| ALDSx  ALDSy | 0. Yes | 1. Yes with some effort | 2. No | 3. Unknown |
| --- | --- | --- | --- | --- |
| 0. Yes | 3. Yes | 2. With some difficulties | 1. With great difficulties | Missing |
| 1. Yes with some effort | 2. With some difficulties | 1. With great difficulties | 1. With great difficulties | Missing |
| 2. No | 1. With great difficulties | 1. With great difficulties | 0. No | Missing |
| 3. Unknown | Missing | Missing | Missing | Missing |

| **Pooled variable** | **MAPT orig. vars.** | **MAPT R2’s** | **FINGER orig. vars** | **FINGER R2’s** | **preDIVA orig. vars** | **preDIVA R2’s** |
| --- | --- | --- | --- | --- | --- | --- |
| able to prepare food (3 values) | ADCS6: Did you prepare meals or snacks? | 0.98 (DtD) | Preparing food | 0.89 (DtD) | ADLS14: Fry an egg and put in on a sandwich;  ALDS18: Peel and cut an apple into pieces | 0.93 (MtS) |
| able/independent to eat (3 values) | ADL6: Feeding (independence for eating; help needed with cutting meat, peeling fruits; dependent) | 1 (DtD) | Eating | 1 (DtD) | NA | - |
| able/independent to go to the toilet (3 values) | ADL3: Toileting (independence for going to the toilet, undressing and getting dressed; needs to be accompanied or helped with getting dressed or undressing; cannot go to the toilet independently) | 1 (DtD) | Function at the toilet | 0.91 (DtD) | ADLS22: Go to the toilet | 1 (DtD) |
| able/independent to wash (3 values) | ADL1: Bathing (independence; partial help; dependent) | 1 (DtD) | Washing up | 0.96 (DtD) | ALDS13: Take a shower | 1 (DtD) |
| able to cut toe nails (3 values) | NA |  | Cutting toe nails | 0.92 (DtD) | ADLS07: Cut your toenails | 1 (DtD) |
| able/independent to get dressed (3 values) | ADL2: Dressing (independence for choosing clothes and getting dressed; independence for choosing clothes and getting dressed, but help needed for putting shoes on; dependent) | 1 (DtD) | Dressing | 1 (DtD) | ALDS20: Put on a pair of trousers; ALDS21: Put on and take off a coat | 0.92 (MtS) |
| able to climb the stairs (3 values) | NA | - | Climbing the stairs | 0.92 (DtD) | ALDS10: Walk up a staircase | 1 (DtD) |
| able to use (own car or) public transportation (3 values) | ADCS2: Did you drive a car without getting lost, or did you travel to wherever you needed by using public or other transport? | 0.97 (DtD) | Using public transportation | 0.92 (DtD) | ALDS05: Travel by bus, tram or metro | 1 (DtD) |
| able to go shopping (3 values) | ADCS5: Did you select and pay for items when shopping? | 1 (DtD) | Shopping | 0.94 (DtD) | ALDS04: Do groceries for several days in one go. | 1 (DtD) |
| able to do light household work (3 values) | NA | - | Light household work | 0.91 (DtD) | ALDS12: Change the bed;  ALDS17: Clear the table after having a meal | 0.89 (MtS) |
| able to do heavy household work (3 values) | NA | - | Heavy household work | 0.91 (DtD) | ALDS03: Vacuum and shove aside furniture | 1 (DtD) |
| able to do the laundry (3 values) | ADCS4: Did you do the laundry? | 1 (DtD) | Washing the laundry | 0.89 (DtD) | NA | - |
| able to manage finances (3 values) | ADCS1: Did you balance a checkbook or credit card statement, or pay bills, or use and ATM to remove or deposit money? | 0.97(DtD) | Managing financials | 0.93 (DtD) | NA | - |
| able to take (and dose) medication (3 values) | ADCS13: Did you take medication regularly – including prescription or over the counter medications? | 0.97 (DtD) | Taking and dosing medication | 0.96 (DtD) | NA | - |
| able to use the phone (3 values) | ADCS12: How well did you make phone calls, including looking up numbers or calling directory assistance if necessary? | 0.95 (DtD) | Using the phone | 0.91 (DtD) | NA | - |
| able to prepare food (4 values) | ADCS6: Did you prepare meals or snacks? | 1 (DP) | Preparing food | 0.99 (DtD) | ADLS14: Fry an egg and put in on a sandwich;  ALDS18: Peel and cut an apple into pieces | 0.93 (MtS) |
| able to do light household work (4 values) | NA | - | Light household work | 0.99 (DtD) | ALDS12: Change the bed;  ALDS17: Clear the table after having a meal | 1 (MtS) |
| able to do the laundry (4 values) | ADCS4: Did you do the laundry? | 1 (DP) | Washing the laundry | 0.98 (DtD) | NA | - |
| able to manage finances (4 values) | ADCS1: Did you balance a checkbook or credit card statement, or pay bills, or use and ATM to remove or deposit money? | 1 (DP) | Managing financials | 1 (DtD) | NA | - |
| able to take (and dose) medication (4 values) | ADCS13: Did you take medication regularly – including prescription or over the counter medications? | 1 (DP) | Taking and dosing medication | 1 (DtD) | NA | - |
| able to use the phone (4 values) | ADCS12: How well did you make phone calls, including looking up numbers or calling directory assistance if necessary? | 1 (DP) | Using the phone | 1 (DtD) | NA | - |

**3. Identification variables**

| **Pooled variable** | **MAPT** | **FINGER** | **preDIVA** |
| --- | --- | --- | --- |
| ID | DP | DP | DP |
| Study (study names abbreviated) | M | F | P |
| Studycenter number | DP | DP | DP |
| Randomisation group (0 = control; 1 = intervention) | DP | DP | DP |
| Visit date | DP | DP | DP |
| Randomisation date | DP | DP | DP |
| Visit index number | DP | DP | DP |

**4. Cardiovascular lifestyle**

| **Pooled variable** | **MAPT orig. vars.** | **MAPT R2’s** | **FINGER orig. vars** | **FINGER R2’s** | **preDIVA orig. vars** | **preDIVA R2’s** |
| --- | --- | --- | --- | --- | --- | --- |
| Current smoking status: |  | 0.96 (MtS) |  | 0.97 (MtS) |  | 1 (DP) |
| - *Never smoked* | - *Currently smoking = ‘no’ and when quitted? = ‘never smoked’* |  | - *Ever smoked = ‘no’ and ever regularly smoked = ‘no’* |  |  |  |
| - *Smoked but quitted* | - *Currently smoking = ‘no’ and when quitted? = ‘less than 3 years ago’ / ‘more than 3 years ago* |  | - *Ever smoked = ‘yes’ and ever regularly smoked = ‘yes’ and currently smokes = ‘not at all’* |  |  |  |
| - *Currently smokes* | - *Currently smoking = ‘yes’* |  | - *Ever smoked = ‘yes’ and ever regularly smoked = ‘yes’ and currently smokes = ‘yes, daily’ / ‘yes, occasionally’* |  |  |  |
| Duration since quitted smoking: | DP | 1 | NA | - | MtS | 0.10 |
| - *Less than 3 years* |  |  |  |  | *Quitted smoking and cessation years < 3* |  |
| - *3 years or more* |  |  |  |  | *Quitted smoking and cessation years >= 3* |  |
| Number of smoking years | NA | - | If ever smoked = ‘yes’ and regularly smoked = ‘yes’: number of smoking years;  Else: missing | 1 (MtS) | If currently smokes or quitted smoking: number of smoking years;  Else: missing | 1 (MtS) |
| Number of cigarettes and other tobacco types per day | NA | - | If ever smoked = ‘yes’ and regularly smoked = ‘yes’: number of manufactured cigarettes + self-rolled cigarettes + pipefulls + cigars per day;  Else: missing | 1 (MtS) | If currently smokes or quitted smoking : number of cigarettes + other tobaccos per day;  Else: missing | 1 (MtS) |
| Alcohol use: | DP | 1 | MtS | 0.80 | DP | 1 |
| - *yes* |  |  | - *alcohol frequency > once per month* |  |  |  |
| - *no* |  |  | - *alcohol*   *frequency < once per month* |  |  |  |
| Weekly number of alcohol drinks | 7*(number of glasses wine + glasses beer + glasses aperitifs + glasses liquors per day) | 1 (MtS) | If alcohol frequency > once per month: number of beers + long drinks + spirits or other strong alcohol measures + glasses wine + strong ciders in the last week;  Else: missing | 1 (MtS) | If alcohol use = ‘yes’ and alcohol frequency = ‘monthly’: alcohol frequency / 4.35  If alcohol use = ‘yes’ and alcohol frequency = ‘weekly‘: DP  Else: missing | 0.98 (MtS) |
| Number of fish dishes per week | NA | - | DP | 1 | If fish consumption = ‘yes’ and fish frequency = ‘monthly’: fish frequency / 4.35  If fish consumption = ‘no’ and fish frequency = ‘weekly‘: DP  Else: 0 | 1 (MtS) |
| Fruit intake: | NA | - | DP | 1 | MtS | 0.95 |
| - *6 portions or more per day* |  |  |  |  | - *Monthly fruit intake >= 168 or weekly fruit intake >= 39* |  |
| - *3-5 portions per day* |  |  |  |  | - *Monthly fruit intake 77-167 or weekly fruit intake 18-38* |  |
| - *1-2 portions per day* |  |  |  |  | - *Monthly fruit intake 29-76 or weekly fruit intake 7-17* |  |
| - *4-6 portions a week* |  |  |  |  | - *Monthly fruit intake 16-28 or weekly fruit intake 4-6* |  |
| - *1-3 portions a week* |  |  |  |  | - *Monthly fruit intake 5-15 or weekly fruit intake 1-3* |  |
| - *Less than 1 portion a week or none* |  |  |  |  | - *Fruit consumption is ‘no’ or monthly fruit intake <= 4* |  |

**5. Medical history**

| **Pooled variable** | **MAPT orig. vars.** | **MAPT R2’s** | **FINGER orig. vars** | **FINGER R2’s** | **preDIVA orig. vars** | **preDIVA R2’s** |
| --- | --- | --- | --- | --- | --- | --- |
| Diabetes (yes/no) | DP | 1 | DP | 1 | DP | 1 |
| Hypertension (yes/no) | DP | 1 | DP | 1 | NA | - |
| History of stroke (yes/no) | DP | 1 | DP | 1 | DP | 1 |
| Hypercholesterolemia (yes/no) | DP | 1 | DP | 1 | NA | - |
| History of cardiovascular disease (yes/no) | DP | 1 | DP | 1 | DP | 1 |
| History of angina pectoris (yes/no) | DP | 1 | DP | 1 | NA | - |
| History of myocardial infarction (yes/no) | DP | 1 | DP | 1 | NA | - |
| History of vascular surgery (yes/no) | DP | 1 | DP | 1 | NA | - |
| Family history of dementia: | DP | 1 | MtS | 1 | MtS | 0.92 |
| - *No* |  |  | - *No family history of dementia* |  | - *Family history of dementia for mother = ‘no’ and family history of dementia for father = ‘no’ and family history of dementia for siblings = ‘no’ or family history of dementia for children = ‘no’* |  |
| - *Yes* |  |  | - *At least one family member with dementia* |  | - *Family history of dementia for mother = ‘yes’ or family history of dementia for father = ‘yes’ or family history of dementia for siblings = ‘yes’ or family history of dementia for children = ‘yes’* |  |
| Family history of dementia among parents or siblings: | MtS | 1 | MtS | 0.84 | MtS | 0.92 |
| - *No* | - *Family history of dementia = ‘yes’ and family history of dementia for one parent = ‘no’ or and family history for two parents = ‘no’ and*   *family of dementia for a sibling = ‘no’* |  | - *No family history of dementia or family history of dementia not among siblings or parents* |  | - *Family history of dementia for mother = ‘no’ and family history of dementia for father = ‘no’ and family history of*   *dementia for siblings = ‘no’* |  |
| - *Yes* | - *Family history of dementia = ‘yes’ and family history of dementia for one parent = ‘yes or family history of dementia for two parents = ‘yes’ or family of dementia for a sibling = yes’* |  | - *Family history of dementia among siblings or parents* |  | - *Family history of dementia for mother = ‘yes’ or family history of dementia for father = ‘yes’ or family history of dementia for siblings = ‘yes’* |  |
| Family history of dementia among parents: | MtS | 1 | NA |  | MtS | 1 |
| - *No* | - *Family history of dementia = ‘yes’ and family history of dementia for one parent = ‘no’ and family history for dementia for two parents = ‘no’* |  |  |  | - *Family history of dementia for mother = ‘no’ and family history of dementia for father = ‘no’* |  |
| - *Yes* | - *Family history of dementia = ‘yes’ or family history of dementia for one parent = yes’ or family history for dementia for two parents = ‘yes’* |  |  |  | - *Family history of dementia for mother = ‘yes’ or family history of dementia for father = ‘yes’* |  |
| Family history of dementia among siblings (yes/no) | DP | 1 | NA | - | DP | 1 |
| Family history of diabetes: | DP | 1 | NA | - | MtS | 0.84 |
| - *No* |  |  |  |  | - *Family history of diabetes for mother = ‘no’ and family history of diabetes for father = ‘no’ and family history of diabetes for siblings = ‘no’ or family history of diabetes for children = ‘no’* |  |
| - *Yes* |  |  |  |  | - *Family history of diabetes for mother = ‘yes’ or family history of diabetes for father = ‘yes’ or family history of diabetes for siblings = ‘yes’ or family history of diabetes for children = ‘yes’* |  |
| Family history of heart disease: | DP | 1 | NA | - | MtS | 0.78 |
| - *No* |  |  |  |  | - *Family history of heart disease for mother = ‘no’ and family history of heart disease for for father = ‘no’ and family history of heart disease for for siblings = ‘no’ or family history of heart for children = ‘no’* |  |
| - *Yes* |  |  |  |  | - *Family history of heart disease for mother = ‘yes’ or family history of heart disease for father = ‘yes’ or family history of heart disease for for siblings = ‘yes’ or family history of heart disease for for children = ‘yes’* |  |
| Current antihypertensive treatment: | MsT | 1 | DtD | 0.93 | DP | 1 |
| - *No* | - *Hypertension = ‘yes’ and antihypertensive*   *medication = ‘no’* |  | - *Never on antihypertensive drugs or between 1 week and 6 months ago for the last time* |  |  |  |
| - *Yes* | - *Hypertension = ‘yes’ and antihypertensive medication = ‘yes’* |  | - *Antihypertensive drugs used in the past 7 days* |  |  |  |
| Current statine use: | MtS | 1 | DP | 1 | DP | 1 |
| - *No* | - *Hypercholesterolemia = ‘yes’ and treatment for hypercholesterolemia = ‘no’* |  |  |  |  |  |
| - *Yes* | - *Hypercholesterolemia = ‘yes’ and treatment for hypercholesterolemia = ‘yes’* |  |  |  |  |  |
| Current diabetes treatment: | MtS | 1 | MtS | 1 | MtS | 1 |
| - *No* | - *Current diabetes = ‘yes’ and diabetes medicine 1,2 or 3 ≠ insulin / tablet* |  | - *Current diabetes = ‘yes, type 1’ or ‘yes, type 2’, or ‘yes, don’t know what type’ and current diabetes treatment = ‘no’* |  | - *Insulin treatment for diabetes = ‘no’ and non-insulin treatment for diabetes = ‘no’* |  |
| - *Insulin only* | - *Current diabetes = ‘yes’ and diabetes medicine 1,2 or 3 = insulin not tablet* |  | - *Current diabetes = ‘yes, type 1’ or ‘yes, type 2’, or ‘yes, don’t know what type’ and current diabetes treatment = ‘insulin’* |  | - *Insulin treatment for diabetes = ‘yes’ and non-insulin treatment for diabetes = ‘no’* |  |
| - *Tablet only* | - *Current diabetes = ‘yes’ and diabetes medicine 1,2 or 3 = tablet not insulin* |  | - *Current diabetes = ‘yes, type 1’ or ‘yes, type 2’, or ‘yes, don’t know what type’ and current diabetes treatment = ‘oral medication’* |  | - *Insulin treatment for diabetes = ‘no’ and non-insulin treatment for diabetes = ‘yes’* |  |
| - *Insulin and tablet* | - *Current diabetes = ‘yes’ and diabetes medicine 1,2 or 3 = insulin and tablet* |  | - *Current diabetes = ‘yes, type 1’ or ‘yes, type 2’, or ‘yes, don’t know what type’ and current diabetes treatment = ‘insulin and oral medication’* |  | - *Insulin treatment for diabetes = ‘yes’ and non-insulin treatment for diabetes = ‘yes’* |  |
| Current antiplatelet treatment (yes/no) | NA | - | DP | 1 | DP | 1 |

**6. Neuropsychological assessment**

| **Pooled variable** | **MAPT orig. vars.** | **MAPT R2’s** | **FINGER orig. vars** | **FINGER R2’s** | **preDIVA orig. vars** | **preDIVA R2’s** |
| --- | --- | --- | --- | --- | --- | --- |
| CDR1: memory | DP | 1 | DP | 1 | NA | - |
| CDR2: orientation | DP | 1 | DP | 1 | NA | - |
| CDR3: judgement | DP | 1 | DP | 1 | NA | - |
| CDR4: common affairs | DP | 1 | DP | 1 | NA | - |
| CDR5: home hobbies | DP | 1 | DP | 1 | NA | - |
| CDR6: personal care | DP | 1 | DP | 1 | NA | - |
| CDR total score | DP | 1 | DP | 1 | NA | - |
| MMSE items 1-30 | DP | 1 (all 30 items) | NA | - | NA | - |
| MMSE subscores 1-11 | DP | 1 (all 11 subscores) | NA | - | DP | 1 (all 11 subscores) |
| MMSE sum score | DP | 1 | DP | 1 | DP | 1 |
| TMT part A in seconds | DP | 1 | DP | 1 | NA | - |
| TMT part A number of corrections | DP | 1 | DP | 1 | NA | - |
| TMT part B time in seconds | DP | 1 | DP | 1 | NA | - |
| TMT part B number of corrections | DP | 1 | DP | 1 | NA | - |
| GDS items 1-20 | DP | 1 | NA | - | DP | 1 |
| Zung items 1-20 | NA | - | DP | 1 | NA | - |
| Depression: | CtD | 0.63 | CtD | 0.42 | CtD | 0.58 |
| - *No* | - *GDS sum score <=5* |  | - *Zung sum score < 45* |  | - *GDS sum score <=5* |  |
| - *Yes* | - *GDS sum score > 5* |  | - *Zung sum score >= 45* |  | - *GDS sum score > 5* |  |

Abbreviations: CDR = Clinical Dementia Rating [6]; MMSE = Mini-Mental State Examination [7]; TMT = Trail Making Test [8]; GDS = Geriatric Depression Scale-15 [9]; Zung = Zung Depression Self-Rating Scale [10]

**7. Sociodemographic characteristics**

| **Pooled variable** | **MAPT orig. vars.** | **MAPT R2’s** | **FINGER orig. vars** | **FINGER R2’s** | **preDIVA orig. vars** | **preDIVA R2’s** |
| --- | --- | --- | --- | --- | --- | --- |
| Age | DP | 1 | DP | 1 | DP | 1 |
| Date of birth | DP | 1 | DP | 1 | DP | 1 |
| Gender | DP | 1 | DP | 1 | DP | 1 |
| Number of years of formal education | - 3: No formal schooling or primary school - 7: Primary school certificate - 9: Vocational high school (O-levels or equivalent) - 12: Baccalaureat (Senior high school / A-levels) - 14: > Baccalaureat | 0.99 | DP | 1 | - 0: no education - 6: primary school - 10: LBO, MULO, ULO, LTS, MAVO, MMS - 12: MBO, HAVO, HBS, VWO, Gymnasium Lyceum - 15: HBO - 16: University | 0.87 |
| Highest qualification obtained: | DtD | 0.90 | DtD | 0.91 | DtD | 0.84 |
| - *Low* | - *No formal schooling, primary school, primary school certificate* |  | - *Elementary school* |  | - *No education or primary school* |  |
| - *Intermediate* | - *Vocational high school, O-levels or equivalent* |  | - *Middle school or vocational school* |  | - *LBO, MULO, ULO, LTS, MAVO, MMS, MBO, HAVO* |  |
| - *High* | - *>= Baccalaureat (Senior high school/A-levels)* |  | - *Senior high school, upper vocational school, vocational high school, university of applied sciences, academic degree* |  | - *HBS, VWO, Gymnasium Lyceum, HBO, University* |  |
| Single: | DtD | 1 | MtS | 1 | DP | 1 |
| - *No* | - *Married or living at home with partner* |  | - *Married = ‘yes’ or cohabitating = ‘yes’* |  |  |  |
| - *Yes* | - *Single, separated, divorced or widowed* |  | - *Married = ‘no’ and cohabitating = ‘no’ and divorced ≠ no and widowed ≠ no* |  |  |  |
| Marital status: | DP | 1 | MtS | 1 | NA | - |
| - *Married or cohabitating* |  |  | - *Married = ‘yes’ or cohabitating = ‘yes’* |  |  |  |
| - *Single* |  |  | - *Single = ‘yes’ divorced ≠ no and widowed ≠ no* |  |  |  |
| - *Divorced or separated* |  |  | - *Divorced = ‘yes’ and married = ‘no’ and cohabitating = ‘no’ and if (widowed = ‘yes’) number of years divorced > years widowed* |  |  |  |
| - *Widowed* |  |  | - *Widowed = ‘yes’ and married = ‘no’ and cohabitating = ‘no’ and if (divorced = ‘yes’) number of years widowed > years divorced* |  |  |  |
| Employment and retirement status: | DP | 1 | MtS | 0.99 | NA | - |
| - *Working and not retired* |  |  | - *Working = ‘yes’ or parttime work = ‘yes’ or self-employment = ‘yes’ and pension = ‘no’* |  |  |  |
| - *Retired but still working* |  |  | - *Working = ‘yes’ or parttime work = ‘yes’ or self-employment = ‘yes’ and pension = ‘yes’* |  |  |  |
| - *Retired and not working* |  |  | - *Working = ‘no’ or parttime work = ‘no’ or self-employment = ‘no’ and pension = ‘yes’* |  |  |  |
| - *Not working for other reasons* |  |  | - *Unemployed = ‘yes’ or laid off = ‘yes’ or out of work for other reasons = ‘yes’ and pension = ‘no’ and parttime work = ‘no’* |  |  |  |

**8. Missings per variable and study (%)**

| **variable** | **FINGER** | **MAPT** | **PREDIVA** |
| --- | --- | --- | --- |
| adlcook_3v | 1% | 0.4% | 1.1% |
| adlcook_4v | 1% | 0.4% | 1.1% |
| adldress_3v | 0.2% | 0% | 0.5% |
| adldrug_3v | 1.2% | 0.6% | 100% |
| adldrug_4v | 1.2% | 0.6% | 100% |
| adleat_3v | 0.2% | 0% | 100% |
| adlheavyhw_3v | 1.1% | 100% | 2.5% |
| adllaundry_3v | 1.4% | 0.4% | 100% |
| adllaundry_4v | 1.4% | 0.4% | 100% |
| adllighthw_3v | 0.4% | 100% | 2.9% |
| adllighthw_4v | 0.4% | 100% | 2.9% |
| adlmoney_3v | 0.5% | 0.3% | 100% |
| adlmoney_4v | 0.5% | 0.3% | 100% |
| adlnail_3v | 0.3% | 100% | 2% |
| adlphone_3v | 0.2% | 0.5% | 100% |
| adlphone_4v | 0.2% | 0.5% | 100% |
| adlshop_3v | 0.2% | 0.3% | 2.2% |
| adlstair_3v | 0.3% | 100% | 0.7% |
| adltransport_3v | 0.3% | 0.4% | 6.6% |
| adlwash_3v | 0.3% | 0% | 0.7% |
| adlwc_3v | 0.2% | 0% | 0.6% |
| age | 0% | 0% | 2.4% |
| alc | 0.6% | 54.9% | 0.5% |
| alcnum | 35.7% | 76.2% | 31.1% |
| anginapectoris | 0.6% | 54.9% | 100% |
| antiplatelet | 1.4% | 100% | 0% |
| ApoA1 | 0.3% | 100% | 7.1% |
| ApoB | 0.3% | 100% | 7.1% |
| birthdate | 0% | 0% | 0% |
| BMI | 0.9% | 0.4% | 0.1% |
| CDR_total | 100% | 0.1% | 100% |
| CDR1 | 100% | 0.1% | 100% |
| CDR2 | 100% | 0.1% | 100% |
| CDR3 | 100% | 0.1% | 100% |
| CDR4 | 100% | 0% | 100% |
| CDR5 | 100% | 0% | 100% |
| CDR6 | 100% | 0% | 100% |
| center | 0% | 0% | 0% |
| chol | 0.3% | 56% | 2.1% |
| creatinine | 0.3% | 55.4% | 2% |
| CRP | 0.3% | 100% | 3.7% |
| depression | 10.6% | 0.5% | 6.1% |
| diab | 0.6% | 54.9% | 0% |
| diabdrug | 87% | 96.8% | 18.4% |
| diaBP | 0.7% | 0.7% | 0.3% |
| educ | 1.3% | 2.1% | 1% |
| eduy | 0.2% | 2.1% | 1% |
| empl | 0.3% | 25.2% | 100% |
| famdementia | 5.2% | 34.5% | 0% |
| famdementia_direct | 5.3% | 76.7% | 0% |
| famdementia_parent | 100% | 76.7% | 0% |
| famdementia_sibling | 100% | 76.7% | 0% |
| famdiab | 100% | 55.3% | 0% |
| famheart | 100% | 55% | 0% |
| fishnum | 0.6% | 100% | 10% |
| fruitnum | 0.2% | 100% | 1.6% |
| gender | 0% | 0% | 0% |
| glycemia_casual | 100% | 86.4% | 2.1% |
| glycemia_fasting | 0.2% | 72.7% | 100% |
| glycemia_normal | 0.2% | 59.1% | 2.1% |
| HDL | 0.3% | 56.1% | 1.9% |
| heart | 0.6% | 54.9% | 0.7% |
| height | 0.9% | 0.2% | 0% |
| highBP | 0.6% | 54.9% | 100% |
| highBPdrug | 45.8% | 77.5% | 0.1% |
| highchol | 0.8% | 54.9% | 100% |
| highcholdrug | 3.4% | 80% | 0.2% |
| homesupport | 93.3% | 0% | 100% |
| LDL | 0.3% | 56.1% | 9.8% |
| marital | 11.6% | 25.3% | 100% |
| MMSE_total | 0.2% | 0% | 0.2% |
| myocinf_hist | 0.5% | 54.9% | 100% |
| pulse | 1.2% | 0.8% | 72.6% |
| randodt | 0% | 0% | 0% |
| random | 0% | 0% | 0% |
| single | 0.6% | 25.3% | 16.3% |
| smoke | 1% | 56.8% | 0.2% |
| smokecess3y | 100% | 82.2% | 45.9% |
| smokenum | 59.6% | 100% | 38.8% |
| smokey | 59.2% | 100% | 38.5% |
| stroke | 0.7% | 55% | 1.3% |
| sysBP | 0.7% | 0.7% | 0.3% |
| TMTA_errors | 0.1% | 0.2% | 100% |
| TMTA_time | 0.2% | 0.2% | 100% |
| TMTB_errors | 6.1% | 2.3% | 100% |
| TMTB_time | 9.2% | 2.1% | 100% |
| trigly | 0.3% | 56% | 2% |
| vascsurg | 0.6% | 54.9% | 100% |
| visitdt | 0% | 0% | 0% |
| visitIndex | 0% | 0% | 0% |
| waist | 0.8% | 67% | 0.3% |
| weight | 0.7% | 0.3% | 0% |

**9. Syntax simulation study**

#check current time

t1 <- Sys.time()

#determine width and height of figure

png(width = 1458, height = 558)

#create function to generate r2s from linear models

sim_r2_func <- function(minindep1, maxindep1, size1, minindep2, maxindep2, size2) {

deps1 <- rep(0, size1)

deps2 <- rep(1, size2)

deps <- c(deps1, deps2)

indeps1 <- sample(minindep1:maxindep1, size1, T)

indeps2 <- sample(minindep2:maxindep2, size2, T)

indeps <- c(indeps1, indeps2)

mod <- lm(deps ~ indeps)

r2 <- summary(mod)$r.squared

r2

}

#vectorize function

sim_r2_func <- Vectorize(sim_r2_func)

#create data for simulation 1a

df1a <- expand.grid(minindep1 = 0,

maxindep1 = 1000,

size1 = 1000,

minindep2 = 1001,

maxindep2 = c(round(1000/34:2), 1000, 1000 * 2:34) + 1001,

size2 = 1000)

#set.seed

set.seed(42)

#calculate r2s

df1a$r2 <- rowMeans(replicate(100, sim_r2_func(df1a$minindep1, df1a$maxindep1, df1a$size1, df1a$minindep2, df1a$maxindep2, df1a$size2)))

#add variable on magnitude difference of the range between set1 and set2

df1a$magnitude_difference <- c(-33:33)

#create data for simulation 1b

df1b <- expand.grid(minindep1 = 0,

maxindep1 = 1000,

size1 = 1000,

minindep2 = 1001,

maxindep2 = 2001,

size2 = c(round(1000/34:2), 1000, 1000 * 2:34))

#set.seed

set.seed(42)

#calculate r2s

df1b$r2 <- rowMeans(replicate(100, sim_r2_func(df1b$minindep1, df1b$maxindep1, df1b$size1, df1b$minindep2, df1b$maxindep2, df1b$size2)))

#add variable on magnitude difference of the sample size between set1 and set2

df1b$magnitude_difference <- c(-33:33)

#get summary statistics for simulation 1a

round(summary(df1a$r2), 2)

#get summary statistics for simulation 1b

round(summary(df1b$r2), 2)

#rbind df1a and df1b data in a single dataframe

df1 <- rbind(df1a, df1b)

#add variable on type of simulation

df1$sim <- factor(rep(1:2, each = 67), labels = c("Range difference", "Sample size difference"))

#load ggplot package

library(ggplot2)

#create left plot

g1ab <- ggplot(df1, aes(x = magnitude_difference, y = r2, color = sim, shape = sim)) +

geom_point() +

ylim(c(0,1)) +

xlab("The factor by which the range or sample size of set 2 differs from set 1") +

ylab("R2") +

labs(color = "Simulation type", shape = "Simulation type") +

theme(axis.title = element_text(face = "bold"),

legend.title = element_text(face = "bold")) +

ggtitle("R-squareds for simulation types 1a and 1b")

#create data for simulation 2

df2 <- expand.grid(minindep1 = 0,

maxindep1 = 1000,

size1 = 1000,

minindep2 = 1001,

maxindep2 = c(round(1000/34:2), 1000, 1000 * 2:34) + 1001,

size2 = c(round(1000/34:2), 1000, 1000 * 2:34))

#set.seed

set.seed(42)

#calculate r2s

df2$r2 <- rowMeans(replicate(10, sim_r2_func(df2$minindep1, df2$maxindep1, df2$size1, df2$minindep2, df2$maxindep2, df2$size2)))

#add variable on range difference of the sample size between set1 and set2

df2$magnitude_difference_maxindep2 <- seq(-33, 33, 1)[match(df2$maxindep2, sort(unique(df2$maxindep2)))]

#add variable on magnitude difference of the sample size between set1 and set2

df2$magnitude_difference_size2 <- seq(-33, 33, 1)[match(df2$size2, sort(unique(df2$size2)))]

#get summary statistics for simulation 2

round(summary(df2$r2), 2)

#create right plot

g2 <- ggplot(df2, aes(x = magnitude_difference_size2, y = magnitude_difference_maxindep2, fill = r2, colour = r2)) +

geom_point() +

scale_color_gradientn(colours = c("firebrick", "green2")) +

scale_fill_gradientn(colours = c("firebrick", "green2")) +

labs(fill = "R2", colour = "R2") +

xlab("The factor by which the sample size of set 2 differs from set 1") +

ylab("The factor by which the range of set 2 differs from set 1") +

theme(axis.title = element_text(face = "bold"),

legend.title = element_text(face = "bold")) +

ggtitle("R-squareds for simulation type 2")

#load gridExtra package

library(gridExtra)

#combine left and right plot in a single plot

grid.arrange(g1ab, g2, nrow = 1)

#export plot as png

dev.off()

#check current time

t2 <- Sys.time()

#show time used for the simulations and the creation of the figure

t2 - t1

**References**

1. Katz, S., et al., *Studies of illness in the aged. the index of adl: a standardized measure of biological and psychosocial function.* Jama, 1963. 185: p. 914-9.

2. Galasko, D., et al., *ADCS Prevention Instrument Project: assessment of instrumental activities of daily living for community-dwelling elderly individuals in dementia prevention clinical trials.* Alzheimer Dis Assoc Disord, 2006. 20(4 Suppl 3): p. S152-69.

3. Holman, R., et al., *The AMC Linear Disability Score project in a population requiring residential care: psychometric properties.* Health Qual Life Outcomes, 2004. 2: p. 42.

4. Lawton, M.P. and E.M. Brody, *Assessment of older people: self-maintaining and instrumental activities of daily living.* Gerontologist, 1969. 9(3): p. 179-86.

5. Kingston, A., et al., *Losing the ability in activities of daily living in the oldest old: a hierarchic disability scale from the Newcastle 85+ study.* PLoS One, 2012. 7(2): p. e31665.

6. Hughes, C.P., et al., *A new clinical scale for the staging of dementia.* Br J Psychiatry, 1982. 140: p. 566-72.

7. Folstein, M.F., S.E. Folstein, and P.R. McHugh, *"Mini-mental state". A practical method for grading the cognitive state of patients for the clinician.* J Psychiatr Res, 1975. 12(3): p. 189-98.

8. Reitan, R.M., *Validity of the Trail Making Test as an Indicator of Organic Brain Damage.* Perceptual and Motor Skills, 1958. 8(3): p. 271-276.

9. Yesavage, J.A. and J.I. Sheikh, *Geriatric Depression Scale (GDS): recent evidence and development of a shorter version.* Clinical Gerontologist, 1986. 5(1-2): p. 165-173.

10. Zung, W.W., *A self-rating depression scale.* Arch Gen Psychiatry, 1965. 12: p. 63-70.
